# New York Odontological Society

**Published:** 1884-06

**Authors:** 


### NEW YORK ODONTOLOGICAL SOCIETY.

A regular monthly meeting of this Society was held on the evening of April 15th, at the residence of Dr. W. H. Dwinelle; the President, Dr. W. Jarvie, Jr., in the chair.

*Dr. N. W. Kingsley*—Exhibited a clamp which he said would apply to anything and everything—a curious piece of mechanism, admirably adapted to all cases. Dr. K. also read a brief paper, which contained a "shy" at the medical profession. It claimed that dentistry, although a specialty, was nevertheless a department of medicine. It had been considered a mere mechanical pursuit, and therefore it had been slighted. Its duties require a delicacy of touch quite equal to any known in general surgery.

Tendencies to caries were partly due to conditions peculiar to ancestors. The direct cause of caries, as with other diseases, is from violation of hygienic laws. Teeth decay from lack of nutrition, or from constitutional derangement, the tendencies to disease being less in healthy than in unhealthy organisms. Caries is due to a solution of lime-salts in the tooth-structure, from acids generated in the mouth. Bacteria are not found beyond the parts decalcified. The food-fermenting acids are in part neutralized by the saliva. The approximal surfaces are the most vulnerable points of attack.

*Dr. J. Smith Dodge, Jr.*—Exhibited two casts, one representing irregularity of the incisors in the mouth of a gentleman forty-six years of age, caused by the loss of molars, which permitted the incisors to come together in such a manner as to force one superior central much forward, and the other much inside its natural position, and thus locking on either side of the inferior incisors when the mouth was closed. An appliance was fitted to the mouth to prevent the incisors from meeting, and with no further effort the teeth within a fortnight fell into a line of regularity (as shown by the second cast), although they had been out of proper position for nearly twenty years.

This case suggested the uncertainty of keeping teeth that have been regulated from returning to their natural state of disorder after retaining plates have been removed, as their natural tendency seems to incline them that way.

*Dr. C. A. Woodward*—Presented a model representing the superior teeth in the mouth of a young lady twenty-one years of age. The left central incisor was missing. The deciduous teeth were extracted many years before, but only one permanent central incisor came, and the vacant space had nearly closed. Wedges were introduced to widen this space, and a few months later four tiny supernumerary teeth made their appearance in a cluster, as shown by the cast. Dr. W. had also a cast showing the mouth of a boy fourteen years of age. Two years before, a sixth-year molar was extracted, and in its place a supernumerary soon after appeared. The attention of the family physician was called by the child's mother to this remarkable case, and after looking at it he stated that he had seen similar cases before, and explained the matter by saying that "in the forcible removal of large teeth little fragments of bone are sometimes left in the socket, and from such fragments these extra teeth are formed"!

*Dr. W. H. Dwinelle*—Spoke of jack-screws for regulating teeth, which he devised and successfully used over twenty years ago. He also cited a case he had under treatment where the patient, a lady, was so exceedingly nervous as to be beyond control. He remarked that with good care and kind treatment such trying patients sometimes turned out the best. He has in some instances of extreme nervousness administered atrophine and morphine.

*Dr. C. F. W. Bodecker*—Exhibited specimens of gold fillings from a dentist in Germany, which were made wholly with burnishers, and in a remarkably brief space of time.

The essayist of the evening, Dr. J. Smith Dodge, Jr., read a paper entitled "The Spheroidal Tendency of Amalgam," in which he proposed to "discuss a theoretical explanation of an undoubted fact"—that almagam fillings which change shape after becoming solid do so from the tendency of amalgam to assume the form of a sphere. This at least seems the most reasonable explanation. He knew not whence it originated, nor had he seen any attempt to prove it. It may have been suggested by familiar facts. When broad, flat-faced fillings are found bulged out at the center and drawn in at the edges, a conclusion is reached that the amalgam is struggling to become a ball. He would not assert this as the veritable theory, but it was one worth considering. He recognized objections to the belief that the molecules of so solid a substance were subject to laws that draw liquids into globular forms, yet as amalgam does alter its shape the molecules must have freedom to change position. As an offset to this theory it may be said that no other solid substance can be produced to demonstrate it. Students of nature are incredulous of "mares' nests," and demand proof concerning unparalleled phenomena. He disclaimed an undoubting belief in this theory, suspecting it may give place to some other less remarkable, but adopts it now as a good working hypothesis. He was led by curiosity to consider the changes referred to in a *geometrical* light, and discovered that such a theory suggested certain definite changes beyond those referred to as "shrinkage." As his mind dwelt on the subject he observed more closely the changes, and found abundant instances of nearly all that had been geometrically deduced. He had this matter in mind for a year, and increased observation convinced him that certain rules could be followed for the formation of amalgam plugs whereby the tendency to a change of form might be utilized to advantage. He proposed to discuss the geometrical theory at first, and then consider facts of practice. "The tendency to spheroidal forms results from mutual attraction of all the molecules as arranged around a common center, and when particles are free to move they naturally go in such direction." "If obstacles interfere they make their nearest possible approach to a sphere, with more or less failure, however, to reach it, the governing law being that every point of the original outline will so change as to approach the outline of a sphere having the same cubical contents." Dr. D. illustrated with diagrams the changes likely to occur in fillings or masses of amalgam of various shapes, in their tendency to assume spheroidal forms. "Let it be carefully observed," said he, "that the lengthening of short diameters is as much a necessary part of the change as the shortening of long ones." He stated also, as a governing law, that where an irregular mass tended to assume a spheroidal form, every point of surface which is more distant from its geometrical center would move towards the center, and every point less distant would move from it; or the long diameters would shorten and the short ones lengthen.

Referring to his diagrams the Doctor explained that where amalgam was placed within an unyielding matrix, like a cavity in a tooth, the filling, being unable to move the walls, would naturally be forced out at the open space and contract at certain points, caused by changes of diameters alluded to. Amalgams in broad, shallow cavities of oblong shape are liable to drop out, from the shortening and thickening of the mass, but where well secured by undercuts at extremities of short diameters, are likely to remain. The principle is that the original form of the amalgam mass determined the changes of its parts, and remembering the rule concerning the changing of diameters, the operator can in many cases secure good results by varying the forms of cavities and plugs.

The Doctor closed by remarking that if the theory suggested was the true one (although he would not positively assert it), he wished to advance certain practical points which he considered valuable. He intimated that "experimental confirmation in practice of theoretical deductions would go far towards establishing the theory suggested." He expressed much interest in his observations, and trusted that what he had stated would awaken others to give the matter reflection and examination.

At the conclusion of the essay, Dr. Dodge stated that he had that day examined teeth previously filled with amalgam, the appearance of which tended to confirm the truthfulness of the theory referred to in his paper.

*Dr. E. A. Bogue*—Experimented with amalgam a number of years ago in various ways. Had witnessed cases similar to those described by Dr. Dodge. Had noticed longitudinal shrinkage in experiments he had made.

*Dr. J. W. Clowes*—Had heard of changes taking place in amalgam fillings, but did not believe that good amalgam would change form. Some poor amalgams were in market, and some contained cadmium. He emphatically condemned cadmium. Formerly he used the old style of fillings made from filings of coin, which were excellent "saving fillings," but became black. To avoid discoloration in some cases, he packed tin in the front of cavities and completed with amalgam. Experimenting in this manner led him to the discovery that tin made amalgam white, and improved its quality. This was in 1845, and he claimed to have been the first who suggested this combination.

*Dr. J. F. P. Hodson*—Stated that Dr. Dodge's paper presented a new phase of the subject, which was interesting. In his own practice he had combined lumps of amalgam already hardened, with fresh amalgam, with a view to prevent the change of shape.

*Dr. B. Lord*—Was much interested in the paper. It gave new light to the subject, and food for thought. He thought it might be the explanation of what he could not understand in his practice with the use of amalgam. He had noticed that cavities filled in different teeth of the same mouth at the same time, and with the same amalgam, exhibited different aspects. "Amalgam fillings do not always fulfill our hopes and expectations in preserving. We find shrinkage and leakage, but more in some cases than in others. It does better in hard teeth." He never feels sure of amalgam. Considers tin better, where favorable conditions exist for its use, but alloy better for some cases, yet finds much difference in alloy.

*Dr. C. E. Francis*—Had also listened with much interest to the paper read by Dr. Dodge, and trusted that it would be well considered by all who were present. He had viewed amalgam as a mystery. In the early days of his practice he was prejudiced against its use, and perhaps then without just cause. He had always used it with some degree of hesitation—not from its liability to discolor, nor from fear of any mercurial effect on the system, but felt it to be unreliable from its tendency to change shape. Whether this change was expansion or contraction had long been a question, but that it frequently receded from the marginal walls of cavities was a fact known to every gentleman present. He had observed that in some cases it would keep cavities well preserved for years, and in other cases would fail in a few months.

*Dr. F. Y. Clark*—Believes amalgam changes shape, hence cannot rely on it, yet there are cases where he feels obliged to use it. In order to overcome shrinkage or expansion he sometimes mixes it with two-thirds its quantity of oxy-phosphate of zinc, which will combine well with it, and finishes by covering the surface with amalgam, to prevent wear.

*Dr. Francis*—Alluded to the combination of amalgam with oxychloride of zinc, which was suggested some years ago by Dr. Wright, of South Carolina. He used it in one or two cases with a fair degree of success.

*Dr. Bogue*—Intimated that "we meet to learn." It disappointed him to feel that so many dentists have no fixed methods for mixing amalgam, and he stated the importance of using a balance for accurately weighing the alloy and mercury. He was first driven to amalgam by necessity, and when he found he *must* use it he investigated concerning it, and tested its merits. It should not be washed, but packed or malleted so hard as to be nearly set. He admitted that there were different grades of amalgam, but considered this a loose way of talking.

*Dr. A. L. Northrop*—Asked if any one was capable of giving a recommendation for amalgam.

*Dr. Lord*—Repeated a remark made by Dr. Francis, that amalgam was a "mystery," as it so varied in appearance and properties.

